# *Mig-6* Gene Knockout Induces Neointimal Hyperplasia in the Vascular Smooth Muscle Cell

**DOI:** 10.1155/2014/549054

**Published:** 2014-12-10

**Authors:** Ju Hee Lee, Sorim Choung, Ji Min Kim, Jung Uee Lee, Koon Soon Kim, Hyun Jin Kim, Jae-Wook Jeong, Bon Jeong Ku

**Affiliations:** ^1^Department of Internal Medicine, Chungnam National University School of Medicine, 282 Munhwa-ro, Jung-gu, Daejeon 301-721, Republic of Korea; ^2^Department of Pathology, Daejeon St. Mary's Hospital, College of Medicine, The Catholic University of Korea, 222 Banpo-daero, Seocho-gu, Seoul 137-701, Republic of Korea; ^3^Department of Obstetrics, Gynecology & Reproductive Biology, Michigan State University, Grand Rapids, MI 49530, USA

## Abstract

Although advances in vascular interventions can reduce the mortality associated with cardiovascular disease, neointimal hyperplasia remains a clinically significant obstacle limiting the success of current interventions. Identification of signaling pathways involved in migration and proliferation of vascular smooth muscle cells (SMCs) is an important approach for the development of modalities to combat this disease. Herein we investigate the role of an immediate early response gene, mitogen-inducible gene-6 (*Mig-6*), in the development of neointimal hyperplasia using vascular smooth muscle specific *Mig-6* knockout mice. We induced endoluminal injury to one side of femoral artery by balloon dilatation in both *Mig-6* knockout and control mice. Four weeks following injury, the artery of *Mig-6* knockout mice demonstrated a 5.3-fold increase in the neointima/media ratio compared with control mice (*P* = 0.04). In addition, *Mig-6* knockout vascular SMCs displayed an increase in both cell migration and proliferation compared with wild-type SMCs. Taken together, our data suggest that *Mig-6* plays a critical role in the development of atherosclerosis. This finding provides new insight into the development of more effective ways to treat and prevent neointimal hyperplasia, particularly in-stent restenosis after percutaneous vascular intervention.

## 1. Introduction

Atherosclerosis remains a leading cause of death and morbidity in developed countries. It is a disease that affects the circulatory system and has distinct clinical manifestations that depend on the particular circulatory bed affected. For example, atherosclerosis of the coronary arteries causes myocardial infarction and angina pectoris. Percutaneous vascular intervention is commonly performed for the treatment of symptomatic atherosclerosis. However, recurrent lumen narrowing occurs in 3–20% of patients despite the introduction of a drug-eluting stent. This limitation presents a clinically significant obstacle responsible for reducing the long-term success of current interventions [1].

Vascular smooth muscle cells (SMCs) are involved in lesion progression, which is responsible for the pathogenesis resulting from atherosclerosis [2]. As part of the vascular response to injury, SMCs migrate from the media, across the intima, into the lumen of the vessel where they proliferate and synthesize cytokines, consequently sustaining the progression of intimal hyperplasia [3–5]. Intimal hyperplasia, in response to mechanical and immunologic insults, is one of the main pathophysiological consequences of postvascular intervention restenosis [5–8]. Therefore, the identification of signaling pathways involved in the migration and proliferation of SMCs during intimal hyperplasia is critical for the development of modalities to combat this disease.

Mitogen-inducible gene-6 (*Mig-6*) is an immediate early response gene that can be induced by various mitogens, stresses, and hormones [9–12].* Mig-6* can interact directly with all four members of the ErbB family, including epidermal growth factor receptor (EGFR) and ErbB2-4, to act as a negative feedback regulator of the ErbB receptor tyrosine kinase pathway [10, 13–15]. Ablation of* Mig-6* in mice leads to the development of epithelial hyperplasia, adenoma, and adenocarcinoma in organs such as the lung, gallbladder, and bile duct [16]. It is well documented as a tumor suppressor gene [12, 16–21] and was recently reported to be involved in cholesterol homeostasis and bile acid synthesis [22]. EGFR is also expressed by vascular SMCs [23, 24], and ligands of EGFR induced cellular proliferation and enhanced migration of vascular SMCs [25–27]. However, the role of* Mig-6* in vascular SMCs has not been evaluated to date.

In this report we investigated the function of MIG-6 in vascular SMCs and its potential role in the development of atherosclerosis and neointima formation. Using a vascular SMC-specific* Mig-6* conditional knockout mouse in an in vivo model of endoluminal injury, together with in vitro SMC cell migration and proliferation assays using small interfering RNA- (siRNA-) mediated* Mig-6* knockdown, we demonstrated a significant effect on SMC migration and proliferation.

## 2. Materials and Methods

### 2.1. Animals

Floxed* Mig-6* (*Mig-6* ^*flox/flox*^) mice were generated as described previously [28]. Transgelin-*Cre* transgenic mice (C57BL/6J) were kindly provided by Dr. Lee from the Korea Research Institute of Bioscience and Biotechnology. Mice were maintained in a controlled environment (12 h light/12 h dark cycle, humidity 50–60%, ambient temperature 23°C ± 1°C) and fed ad libitum. All animal experiments were performed in the animal care facility at the Chungnam National University School of Medicine according to the institutional guidelines for the care and use of laboratory animals. Experimental protocols were approved by the institutional review board of Chungnam National University School of Medicine.

### 2.2. Primary Culture of Vascular Smooth Muscle Cells

Aortas from wild-type and knockout mice were minced and incubated at 37°C in Dulbecco's Modified Eagle's Medium (DMEM). The endothelia and adventitia were stripped away, and then the adventitia-free aorta was cut into 3–5 mm segments. The tissue was incubated for an additional 24 hours in DMEM containing 50% fetal bovine serum (FBS) to isolate SMCs. Cells were further cultured in DMEM containing 10% FBS.

### 2.3. Cell Migration Assay

The transwell migration assay was performed to quantify vascular SMC chemotaxis. A 24-well transwell apparatus (Costar, Corning, NY, USA) was used, with each well containing a 6.5 mm polycarbonate membrane with 0.8 *μ*m pores and coated with 0.1% gelatin. Vascular SMCs were incubated in serum-reduced medium for 24 hours. The cells were trypsinized, resuspended, and plated. The bottom chamber was filled with DMEM containing 0.1% FBS and the indicated chemotactic agent. The chamber was incubated for 1–4 hours at 37°C in a CO_2_ incubator. The membranes were fixed, and the attached cells were stained. The number of cells that had migrated across the membrane was quantified as the number of cells per low-power microscopic field.

### 2.4. Cell Proliferation Assay

For proliferation assays, cells grown to 70% confluence were serum-starved in 0.1% FBS medium for 24 hours and then seeded onto a 96-well plate. Cells were treated with chemotactic agent in DMEM containing 0.1% FBS and incubated for 1–4 hours at 37°C in a CO_2_ incubator. After changing the media, a solution from the cell counting kit-8 (Dojindo, Kumamoto, Japan) was added to the cells. After 2 hours, the absorbance at 450 nm was measured using an Emax Precision Microplate Reader (Molecular Devices, Sunnyvale, CA, USA).

### 2.5. Small Interfering RNA Experiments

For siRNA transfection, human vascular SMCs were incubated with CSC complete recombinant serum-free medium and transfected with nonspecific siRNA and* Mig-6* siRNA using RNAiMAX (Invitrogen, Carlsbad, CA, USA) following the manufacturer's protocol. After 4 hours, the medium was changed, and cells were incubated for a further 48 hours.

### 2.6. Surgical Procedure

Endoluminal injury to the femoral artery was produced using a 0.25 mm diameter balloon angioplasty guidewire (Advanced Cardiovascular Systems, Santa Clara, CA). The animals were anesthetized by intraperitoneal pentobarbital sodium injection (Nembutal, Abbott Laboratories, Chicago, USA), 50 mg/kg body weight. While being viewed under a surgical microscope (Carl Zeiss, Mainz, Germany), a groin incision was made. The femoral artery was clamped temporarily at the level of the inguinal ligament, and an arteriotomy was made distal to the epigastric branch. The guidewire was then inserted, the clamp removed, the wire advanced to the level of the aortic bifurcation, and the balloon inflated. After removal of the wire, the arteriotomy site was ligated. Injured arteries were harvested 4 weeks after balloon dilation.

### 2.7. Histology and Immunohistochemistry

Specimens were perfused-fixed with 4% paraformaldehyde in PBS at 100 mmHg for 15 minutes followed by excision of the femoral artery. Specimens were fixed overnight in 4% paraformaldehyde in PBS and decalcified in 1% formic acid. Two 2 mm thick transverse segments were cut from each artery at the level of injury in the femoral artery and processed for paraffin embedding. Five micrometer sections were cut and stained with Masson's trichrome and hematoxylin-eosin. Human arterial tissue was obtained from patients who underwent vascular surgery. For immunohistochemistry, tissue sections were deparaffinized, rehydrated, and heated in a microwave for 10 minutes in citrate buffer. The tissue sections were then incubated with primary antibodies for 16 hours at 4°C. Immunohistochemistry was performed using a Polink-1 HRP Rat-NM DAB Detection System (GBI Inc., WA, USA).

### 2.8. Western Blot Analysis

Total protein from cultured cells or mouse tissues was extracted in lysis buffer and separated using SDS-PAGE. The separated proteins were transferred to a nitrocellulose (NS) membrane (Amersham Biosciences, Freiburg, Germany). Membranes were blocked with 5% skim milk in TBS and incubated with primary antibodies overnight at 4°C followed by secondary antibodies for 1 hour at room temperature. Immunoreactive bands were developed using ECL reagents. To control for loading, the membrane was stripped and reprobed with mouse-anti-*β*-actin antibody (Sigma-Aldrich, St. Louis, USA). Primary antibodies against mouse Mig-6, phosphorylated EGFR, total EGFR, phosphorylated AKT, total AKT, phosphorylated ERK, and total ERK were purchased from Cell Signaling (Danvers, Massachusetts, USA). The anti-*α*-smooth muscle actin antibody was obtained from Boster Biological Technology (Oxfordshire, UK). Secondary antibodies (goat anti-mouse and goat anti-rabbit) were obtained from Cell Signaling.

### 2.9. Statistical Analyses

Data are presented as means ± or + standard error (SE). Statistical significance for comparisons was determined using Student's two-tailed *t-*test. A *P* value less than 0.05 was considered statistically significant.

## 3. Results

### 3.1. Generation of* Mig-6* Conditional Knockout Vascular Smooth Muscle Using* Tagln* ^*Cre*^

**Figure 1:**
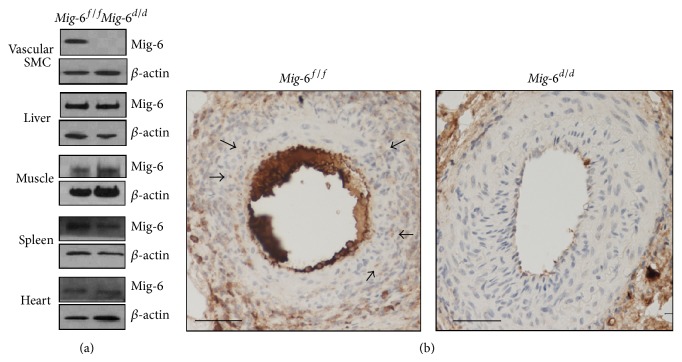
Generation of vascular smooth muscle specific* Mig-6* knockout mice. (a) Western blotting of Mig-6 protein in the various tissues of* Mig-6* ^*f/f*^ and* Mig-6* ^*d/d*^ mice. (b) Mig-6 expression (arrow) was identified by immunohistochemistry in the femoral artery of* Mig-6* ^*f/f*^ and* Mig-6* ^*d/d*^ mice. Scale bar, 100 *μ*m.

In order to investigate the role of* Mig-6* in vascular SMCs, we generated vascular SMC-specific* Mig-6* conditional knockout mice. We crossed floxed* Mig-6* mice (*Mig-6* ^*f/f*^) with mice expressing a* Cre* recombinase gene under the control of the transgelin promoter (*Tagln* ^*Cre*^ *Mig-6* ^*f/f*^;* Mig-6* ^*d/d*^). Smooth muscle specific conditional* Mig-6* ablation was confirmed by Western blot analysis of* Mig-6* protein levels in primary cultures of vascular SMCs and in whole liver, skeletal muscle, spleen, and heart. Protein expression was specifically decreased in the vascular SMCs of* Mig-6* ^*d/d*^ mice compared with* Mig-6* ^*f/f*^ mice (Figure 1(a)). However, we did not observe differences in the expression levels in the liver, skeletal muscle, spleen, or heart between* Mig-6* ^*f/f*^ and* Mig-6* ^*d/d*^ mice. Immunohistochemistry confirmed that the expression of* Mig-6* was decreased in the vascular smooth muscle of* Mig-6* ^*d/d*^ mice (Figure 1(b)).

### 3.2. *Tagln* ^*Cre*^ *Mig-6* Knockout Mice Display Enhanced Neointima Formation after Femoral Arterial Injury

**Figure 2:**
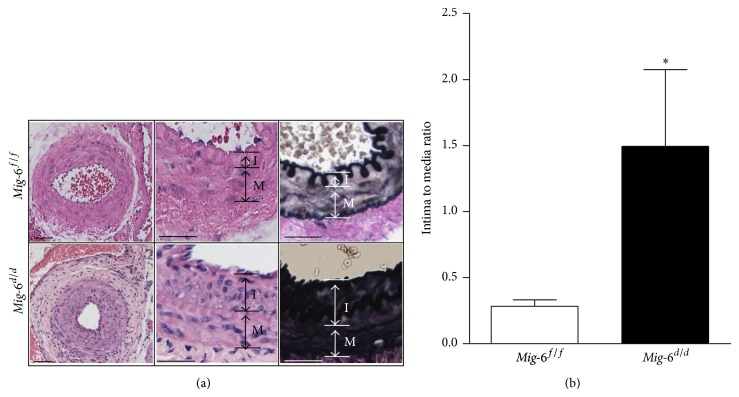
Neointima formation is increased in the vascular smooth muscle specific* Mig-6* knockout mice. (a) Representative cross sections of balloon-catheter injured femoral artery. Left column, hematoxylin and eosin stain ×200; middle column, hematoxylin and eosin stain ×400; right column, Masson's trichrome stain ×400. I, intima; M, media; scale bar, 50 *μ*m. (b) Quantification of intima to media ratio. *n* = 6 for* Mig-6* ^*f/f*^ mice; *n* = 7 for* Mig-6* ^*d/d*^ mice. ^*^ *P* < 0.05.

To ascertain the role of* Mig-6* in the development of atherosclerosis, morphological analysis was performed on the femoral artery of* Mig-6* ^*d/d*^ and* Mig-6* ^*f/f*^ mice following vascular injury. We provided endoluminal injury to one side of the femoral artery using balloon dilatation in* Mig-6* ^*d/d*^ and* Mig-6* ^*f/f*^ mice. Four weeks after injury, both injured and uninjured arteries were dissected and stained with hematoxylin-eosin and Masson's trichrome. No difference in morphology was noted in the uninjured arteries (data not shown). However, the injured arteries of* Mig-6* ^*d/d*^ mice demonstrated an increased neointimal area (Figure 2(a)). This corresponded to a 5.3-fold increase in the neointima/media ratio of* Mig-6* ^*d/d*^ (*n* = 6) compared with* Mig-6* ^*f/f*^ (*n* = 7) vessels (*P* = 0.04) (Figure 2(b)).

### 3.3. EGFR Phosphorylation Was Increased in* Mig-6 *Knockout Vascular SMCs

**Figure 3:**
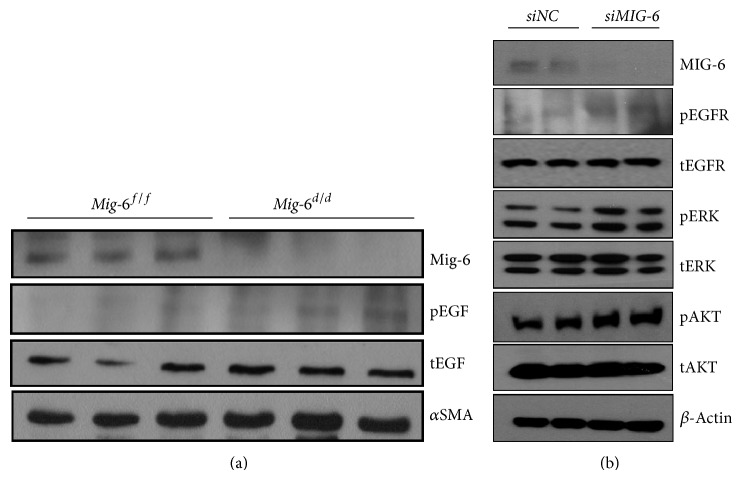
EGFR phosphorylation of vascular SMCs with regard to* Mig-6* knockout. (a) Western blotting for phospho-EGFR in cultured vascular SMCs of* Mig-6* ^*d/d*^ and* Mig-6* ^*f/f*^ mice. pEGFR, phospho-EGFR; *α*SMA, *α*-smooth muscle actin. (b) Western blotting for EGFR and downstream signals according to transfection of siRNA into human vascular SMCs.* siNS*, transfection of nonspecific siRNA;* siMIG-6*, transfection of siRNAs against* MIG-6*; pEGFR, phospho-EGFR; tEGFR, total EGFR; pERK, phospho-ERK; tERK, total ERK; pAAK, phospho-Akt; tAKT, total AKT.

MIG-6 is a known inhibitor of EGFR [14, 15, 29]. To investigate the role of EGFR signaling in the enhanced neointima formation of* Mig-6* ^*d/d*^ mice, vascular SMCs were isolated from the aortas of* Mig-6* ^*d/d*^ and* Mig-6* ^*f/f*^ mice. We observed no difference in total EGFR levels in cultured vascular SMCs from* Mig-6* ^*d/d*^ versus* Mig-6* ^*f/f*^ mice by Western blot. However, phosphorylation of EGFR was increased in* Mig-6* ^*d/d*^ mice compared with* Mig-6* ^*f/f*^ mice (Figure 3(a)). Therefore, as expected, activation of EGFR signaling in vascular SMCs is implicated in enhanced neointima formation observed in* Mig-6* knockout mice. To further explore the effects of* Mig-6* ablation on EGFR signaling in human vascular SMCs, we transfected siRNAs against* Mig-6* into human vascular SMCs.* Mig-6* knockdown in human vascular SMCs resulted in increased phosphorylation of EGFR and EGFR downstream signaling molecules, including ERK and AKT (Figure 3(b)).

### 3.4. Migration and Proliferation Were Increased in Cultured Vascular SMCs from* Mig-6* Knockout Mice

**Figure 4:**
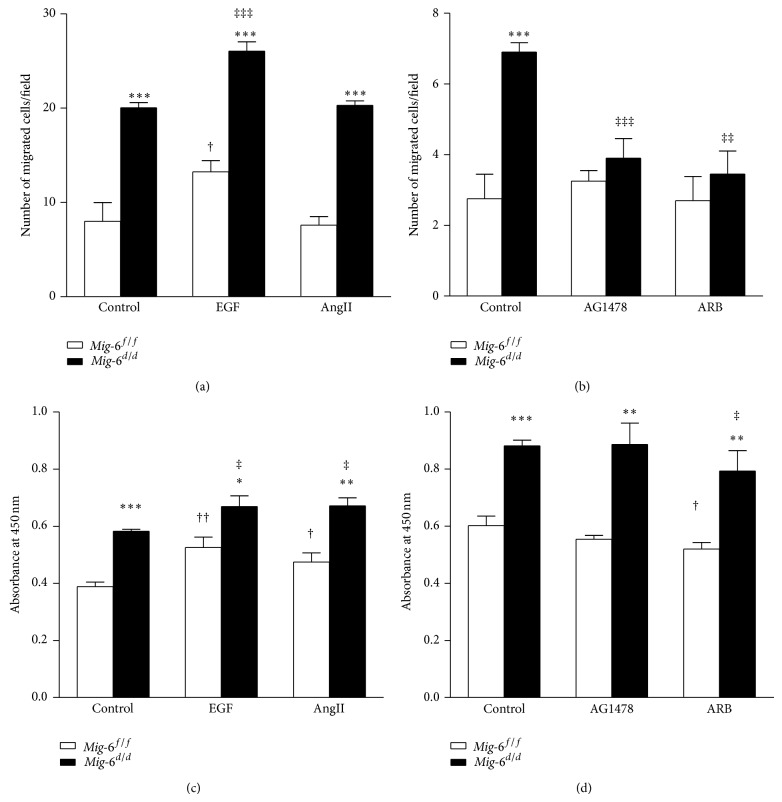
Migration and proliferation of primary cultured vascular SMCs from* Mig-6* ^*d/d*^ and* Mig-6* ^*f/f*^ mice. (a) Changes of cell migration in response to EGFR activators. (b) Changes of cell migration in response to EGFR inhibitors. (c) Changes of cell proliferation in response to EGFR activators. (d) Changes of cell proliferation in response to EGFR inhibitors. *n* = 4, 10~12-week-old male mice. EGF 10 ng/mL, AngII 100 mM, AG1478I 250 nM, and ARB 100 *μ*M. EGF, epidermal growth factor; AngII, angiotensin II; ARB, angiotensin receptor blocker. ^*^ *P* < 0.05, ^**^ *P* < 0.01, and ^***^ *P* < 0.001 versus* Mig-6* ^*f/f*^; ^†^ *P* < 0.05 and ^††^ *P* < 0.01 versus* Mig-6* ^*f/f*^ control; ^‡^ *P* < 0.05, ^‡‡^ *P* < 0.01, and ^‡‡‡^ *P* < 0.001 versus* Mig-6* ^*d/d*^ control.

A key element in the development of the neointima is the proliferation of intimal SMCs and migration of SMCs from the vessel media to the intima [3]. To determine whether enhanced SMC proliferation and migration also occur in vitro, we investigated the behavior of cultured vascular SMCs from* Mig-6* ^*d/d*^ and* Mig-6* ^*f/f*^ mice. First, we examined the migration of SMCs in response to various chemotactic agents. Vascular SMCs from* Mig-6* ^*d/d*^ mice demonstrated a 2.5-fold increase in migration compared with the cells from* Mig-6* ^*f/f*^ mice (*P* < 0.001) (Figure 4(a)). In the presence of epidermal growth factor (EGF), vascular SMCs from both* Mig-6* ^*f/f*^ and* Mig-6* ^*d/d*^ mice demonstrated enhanced migration (1.7-fold, *P* = 0.031, and 1.3-fold, *P* < 0.001, resp.). Therefore, EGF provided an additive effect on cell migration in the* Mig-6* ^*d/d*^ vascular SMCs. EGFR transactivation through G protein-coupled receptors by angiotensin II has also been demonstrated [30–32]. In cultured vascular SMCs, angiotensin II increased migration of* Mig-6* ^*d/d*^ vascular SMCs additively. On the other hand, treatment with AG1478, an EGFR inhibitor, and angiotensin receptor blocker (ARB) abrogated this additive effect in* Mig-6* ^*d/d*^ vascular SMCs (Figure 4(b)).

In addition to enhanced migratory potential, the* Mig-6* knockout vascular SMCs displayed a 1.5-fold increase in proliferation compared with wild-type cells (*P* < 0.001) (Figure 4(c)). The enhanced proliferation was observed in the presence of EGF and also angiotensin II in* Mig-6* ^*d/d*^ vascular SMCs. Conversely, treatment of AG1478 did not reduce proliferation of* Mig-6* ^*d/d*^ vascular SMCs compared with untreated cells (Figure 4(d)).

### 3.5. Migration and Proliferation Were Increased in* Mig-6* Knockout Human Vascular SMCs

**Figure 5:**
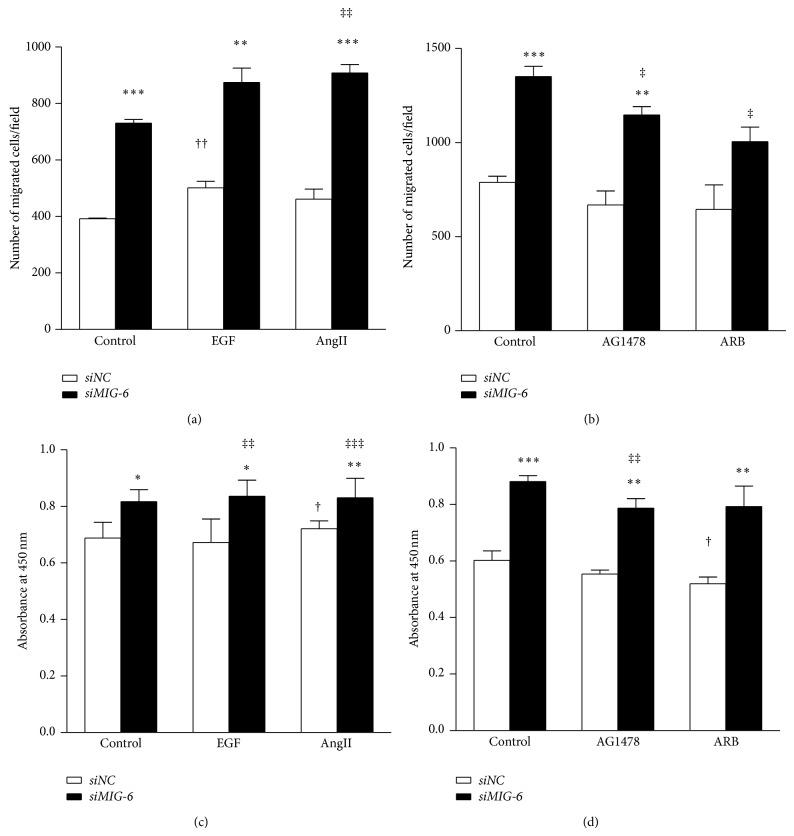
Migration and proliferation of* MIG-6*-silenced human vascular SMCs. (a) Changes of cell migration in response to EGFR activators. (b) Changes of cell migration in response to EGFR inhibitors. (c) Changes of cell proliferation in response to EGFR activators. (d) Changes of cell proliferation in response to EGFR inhibitors. EGF 10 ng/mL, AngII 100 mM, AG1478I 250 nM, and ARB 100 *μ*M. EGF, epidermal growth factor; AngII, angiotensin II; ARB, angiotensin receptor blocker. ^*^ *P* < 0.05, ^**^ *P* < 0.01, and ^***^ *P* < 0.001 versus* Mig-6* ^*f/f*^; ^†^ *P* < 0.05 and ^††^ *P* < 0.01 versus* Mig-6* ^*f/f*^ control; ^‡^ *P* < 0.05, ^‡‡^ *P* < 0.01, and ^‡‡‡^ *P* < 0.001 versus* Mig-6* ^*d/d*^ control.

To ascertain the role of MIG-6 in cell migration and proliferation in human vascular SMCs, we also performed migration and proliferation assays in siRNA-mediated* Mig-6* knockdown human vascular SMCs.* Mig-6* knockdown in human vascular SMCs also demonstrated an increase in migration and proliferation compared with wild-type cells (1.9-fold, *P* < 0.001, and 1.5-fold, *P* < 0.001, resp.) (Figures 5(a) and 5(c)). Treatment of angiotensin II significantly increased migration in* Mig-6* knockout human vascular SMCs (Figure 5(a)). Separate treatment with either EGF or angiotensin II significantly increased proliferation in* Mig-6-*ablated human vascular SMCs (Figure 5(c)). However, treatment with AG1478 or ARB led to decreases in migration and proliferation compared with untreated cells in* Mig-6* knockout human vascular SMCs (Figures 5(b) and 5(d)). Despite the low level of detection of MIG-6 in human vascular SMCs (Figure 3(b)), suggestive of incomplete knockout of* Mig-6*, vascular SMCs exhibited a significant increase in migration, comparable to that of primary cultured vascular SMCs. This indicated that sufficient functional* Mig-6* knockdown had been achieved by siRNA transfection in the vascular SMCs.

## 4. Discussion

All four members of the EGFR family and most of the EGF-like ligands are expressed in vascular smooth muscle cells [33]. Early evidence for a direct role of EGFR in vascular SMC activation was provided by studies in which treatment with EGF promoted bovine vascular SMC proliferation [25]. EGFR has also been demonstrated to mediate both cell proliferation and DNA synthesis in cultured rat aortic smooth muscle cells [23, 24]. More recently, EGFR was found to play an important role in vascular SMC survival and matrix homeostasis using mice with an EGFR-targeted deletion [34].

Herein we investigated the role of EGFR in the proliferation and migration of vascular SMCs by ablating* Mig-6* in both primary cultured murine SMCs and human vascular SMCs. Loss of* Mig-6* enhanced vascular SMC migration, and this effect was inhibited by the addition of either AG1478 or ARB. However, while a loss of* Mig-6* enhanced vascular SMC proliferation, AG1478 and ARB did not produce a biologically significant inhibition. A potential explanation for this observation is that AG1478 may have been administered at a suboptimal dose (250 nM). In other cell lines, particularly tumor cells, higher doses are typically used [35–38]. However, based on other studies using vascular SMCs, 250 nM is considered sufficient [39–41]. Therefore, in summary, increased migration in* Mig-6* knockout vascular SMCs is mediated by EGFR activation. However, increased cellular proliferation appears to be induced directly by the loss of* Mig-6*, although we could not clarify the exact underlying mechanism.

To date, a limited number of studies have examined the role of EGFR in the development of atherosclerosis, despite the expression of EGFR in intimal SMCs within human atherosclerotic plaques [42]. Betacellulin, a member of the EGF family, has also been detected in both intimal and medial SMCs of the aortic wall by immunohistochemical staining. Numerous betacellulin-positive SMCs surrounding the core lesion of atherosclerotic plaques have been observed [42]. Epiregulin, another ligand of EGFR, is also detectable in human atherosclerotic arteries [43]. With regard to restenosis, some promising lines of evidence are being explored in animal models currently. Monoclonal blocking antibodies against EGFR inhibited medial SMC proliferation and intimal hyperplasia in balloon-injured rat carotid arteries [44]. Moreover, periadventitial delivery of an EGFR-blocking monoclonal antibody inhibited neointimal macrophage accumulation and neointimal thickening after balloon-catheter angioplasty in a hypercholesterolemic rabbit [45].

In this study, we demonstrated the role of EGFR signaling in the development of atherosclerosis and restenosis in vivo, using* Mig-6* conditional-knockout mice. Vascular SMCs from* Mig-6* knockout mice demonstrated an increase in EGFR phosphorylation and EGFR downstream signaling. Balloon-injured arteries of* Mig-6* knockout mice presented with increased neointimal area, while there were no significant changes observed in the morphology of uninjured arteries. In this report, we did not confirm the role of EGFR in excessive neointima formation using pharmacological EGFR blocking agents, such as Erlotinib, Lapationib, or Irresa, in vivo. Therefore, the excessive neointima formation could be induced by Mig-6 loss itself, as we noted previously. Similarly, there are also reports on the phenotypes of the breast and endometrium associated with a loss of Mig-6 but unrelated to excessive EGFR signaling [46, 47].

Percutaneous coronary intervention using stenting is widely performed in the treatment of symptomatic coronary disease. Nevertheless, serious concerns exist regarding late complications, such as in-stent restenosis and late stent thrombosis. Although drug-eluting stents have minimized the limitations of bare-metal stents, clinical and histologic studies have demonstrated evidence of continuous neointimal growth during long-term follow-up [40]. The precise mechanisms of neoatherosclerosis development with drug-eluting stents remain unclear to date. Our results demonstrate a potential role for Mig-6 or EGFR signaling in the development of neoatherosclerosis and could represent a novel pathway contributing to disease. Previously, we performed* Mig-6* conditional knockout in the liver using the albumin-*Cre* mouse model [22]. These mice exhibited increased serum levels of low density lipoprotein cholesterol. The genes that regulate lipid metabolism, bile acid, and cholesterol synthesis were also altered in the liver. Taking these two studies together,* Mig-6* clearly plays an important role in the development of atherosclerosis.

In conclusion, we have found that smooth muscle cell-specific ablation of* Mig-6* induced neointimal hyperplasia after balloon injury in the mouse model. In addition,* Mig-6* knockout in vascular SMCs demonstrated increases in cell migration and proliferation potential. To our knowledge, this is the first report of* Mig-6* involvement in the vascular biology of atherosclerosis. Furthermore, this represents a potentially novel mechanism to explain neointimal hyperplasia. This information will provide new insight into aiding the development of more effective strategies to treat and prevent atherosclerosis, specifically in-stent restenosis after percutaneous vascular intervention.
